# *Tbx6* Regulates Left/Right Patterning in Mouse Embryos through Effects on Nodal Cilia and Perinodal Signaling

**DOI:** 10.1371/journal.pone.0002511

**Published:** 2008-06-25

**Authors:** Anna-Katerina Hadjantonakis, Elinor Pisano, Virginia E. Papaioannou

**Affiliations:** 1 Department of Genetics and Development, College of Physicians and Surgeons of Columbia University, New York, New York, United States of America; 2 Developmental Biology Program, Sloan-Kettering Institute, New York, New York, United States of America; Baylor College of Medicine, United States of America

## Abstract

**Background:**

The determination of left/right body axis during early embryogenesis sets up a developmental cascade that coordinates the development of the viscera and is essential to the correct placement and alignment of organ systems and vasculature. Defective left-right patterning can lead to congenital cardiac malformations, vascular anomalies and other serious health problems. Here we describe a novel role for the T-box transcription factor gene *Tbx6* in left/right body axis determination in the mouse.

**Results:**

Embryos lacking *Tbx6* show randomized embryo turning and heart looping. Our results point to multiple mechanisms for this effect. First, *Dll1*, a direct target of Tbx6, is down regulated around the node in *Tbx6* mutants and there is a subsequent decrease in nodal signaling, which is required for laterality determination. Secondly, in spite of a lack of expression of *Tbx6* in the node, we document a profound effect of the *Tbx6* mutation on the morphology and motility of nodal cilia. This results in the loss of asymmetric calcium signaling at the periphery of the node, suggesting that unidirectional nodal flow is disrupted. To carry out these studies, we devised a novel method for direct labeling and live imaging cilia in vivo using a genetically-encoded fluorescent protein fusion that labels tubulin, combined with laser point scanning confocal microscopy for direct visualization of cilia movement.

**Conclusions:**

We conclude that the transcription factor gene *Tbx6* is essential for correct left/right axis determination in the mouse and acts through effects on notch signaling around the node as well as through an effect on the morphology and motility of the nodal cilia.

## Introduction

The bilateral, mirror-image symmetry that characterizes the vertebrate body is little more than skin deep, as many internal organs such as the heart, gut, liver and lungs are asymmetric. The development of left/right (L/R) asymmetry involves the initial breaking of bilateral symmetry by the generation of an asymmetric signal in or near the embryonic node followed by the transfer of information from the node to the left lateral plate mesoderm (LPM). This induces a signaling cascade in the left LPM, and the transformation of L/R asymmetric signals into left- or right-specific morphogenetic programs in the visceral organ primordia [1]–[5]. In the mouse, the first genes known to be asymmetrically expressed are *Nodal* around the node, followed by *Nodal, Lefty2*, and *Pitx2* in the left LPM and *Lefty1* in the left floor plate of the neural tube [6]. Morphological asymmetry appears first in the heart where a leftward displacement of the future atrioventricular canal presages the eventual dextral looping of the heart tube [7]. Shortly thereafter, the embryo undergoes axial rotation toward its right side. Additional morphological asymmetries appear during development of organs, culminating in a highly asymmetric visceral arrangement in the adult.

An early clue to the initial symmetry-breaking event came from observations that human patients with defects in dynein, a component of the ciliary motor, exhibit heterotaxia [8] as do mice carrying *inversus viscerum* (*iv*), a mutation in the gene encoding left-right dynein (*lrd*) [9]. Mutations in several other genes that affect cilia also cause laterality defects, notably, *polycystin-2* (*pkd*2) [10], which codes for a calcium (Ca^2+^) channel associated with cilia and thought to play a role as a mechanosensor, and *polaris*, which is involved in ciliary assembly and may have a role in intraflagellar transport [11]. Use of morpholinos in zebrafish provided direct evidence that *pkd2* and *polaris* control L/R determination through effects on ciliated cells in the node [12].

Recent work has shown that the posteriorly tilted cilia in the ventral node beat with a clockwise rotational movement that sets up a leftward flow of extracellular fluid, known as the nodal flow. The reversal of nodal flow can reverse laterality [13] and an artificial fluid flow across the node can rescue the laterality defects in *iv* mutants, which have immotile cilia, as well as *inversion of embryonic turning* (*inv*) mutants, which have weakly motile cilia [13]–[15]. Thus nodal flow is the key symmetry-breaking event responsible for the establishment of laterality differences [14], [16]–[18].

There are two prevailing models regarding how nodal flow causes a break in symmetry and generates a laterality signal: the first, known as the two-cilia model, proposes that leftward nodal flow generated by motile cilia is sensed by nonmotile mechanosensory cilia at the periphery of the node, causing asymmetric Ca^2+^ signaling that triggers a lateralized gene regulatory cascade [19], [20]. The second model, known as the morphogen gradient model, proposes that an extracellular morphogen is transported leftward by nodal flow creating a gradient [17]. A variation on this model has recently been proposed [21] based on the observation of a novel mode of extracellular transport of morphogens via nodal vesicular parcels (NVPs). NVPs are released from microvilli on the surface of nodal cells and propelled toward the left wall of the node by the nodal flow where they fragment and release their contents. The vesicles have been suggested to transport sonic hedgehog (SHH) and retinoic acid (RA), thus potentially providing morphogens to the left side of the node and culminating in the asymmetric activation of Ca^2+^ signaling [21]. However, unlike the situation in the chick, there is as yet no evidence for asymmetric hedgehog signaling in the node of the mouse [22] and putative morphogens responsible for symmetry breaking have not been identified.

The transforming growth factor-β (TGFβ) family member *Nodal* is a key determinant of laterality and is involved in L/R determination at several levels [2]. In mice, *Nodal* is initially expressed symmetrically the periphery of the node, with a transient, higher level of expression developing on the left side [23], [24]. By the 3–4-somite stage, it is also expressed in the left LPM in an asymmetric pattern that is conserved in all vertebrates. How *Nodal* expression in the node translates into asymmetric *Nodal* expression in the LPM is unknown, although recent work indicates that the signal may be relayed through the mesoderm [25]. Loss or reduction of perinodal *Nodal* results in lack of expression of *Nodal*, *Lefty2*, and *Pitx2* in the LPM [26]–[28].

Several studies show that Notch signaling is directly upstream of *Nodal* expression [29]–[32]. Embryos mutant for the Notch ligand *Delta-like 1* (*Dll1*), the primary mediator of Notch signaling *RBPjk*, or doubly mutant for the receptors *Notch1* and *Notch2* have randomized laterality. Furthermore, node-specific expression of *Nodal* can be eliminated by mutation of RBP-J binding sites in the *Nodal* promoter [29]. Recently, *Wnt3a* has also been implicated early in the genetic hierarchy of laterality determination. Mutants for *Wnt3a* show randomized heart looping and turning, and expression of *Nodal*, *Lefty2*, and *Pitx2* are all delayed and then expressed bilaterally, whereas *Lefty1* is not expressed. In addition, reduced expression of polycystin-1 (PC1) in nodal cilia, a protein that interacts with polycystin-2 (PC2), indicates a potential role for *Wnt3a* in monocilia. It was postulated that WNT signaling might play dual roles in laterality determination by regulating Notch signaling through *Dll1*, as well as affecting nodal cilia through PC1 [33]. However, another study reported a lack of PC1 in nodal cilia and the lack of a laterality phenotype in *Pkd1* mutant mice [34].

*Tbx6* is a T-box transcription factor gene that has important roles in the specification of presomitic mesoderm (PSM) and the formation of somite borders [35]–[39]. Studies with the hypomorphic *Tbx6^rv^* allele have revealed an interaction between *Tbx6* and *Dll1* in the PSM [40], [41]. Furthermore, *Tbx6* acts synergistically with WNT signaling to regulate *Dll1* expression in PSM [42], raising the possibility of involvement of *Tbx6* in laterality determination through regulation of *Dll1*. We have investigated laterality in *Tbx6* mutant embryos and have observed heterotaxia in the homozygous null mutants, which has not been previously reported. Here we explore the mechanism of this laterality defect and show that *Tbx6* is upstream of the Notch signaling pathway in the perinodal region. In addition, *Tbx6* has profound effects on the morphology and motility of nodal cilia that result in a disruption of asymmetric Ca^2+^ signaling around the node.

## Materials and Methods

### Mice and embryo collection

The null allele, *Tbx6^tm1Pa^* [37] and a null expression reporter allele, *Tbx6^tm2Pa^*, which deletes exon 2, part of exon 3 and has an *H2B-EYFP* fusion gene [43], [44] inserted in frame into exon 1, were used in these studies. These alleles produce indistinguishable homozygous phenotypes (our unpublished data). They were maintained on mixed genetic backgrounds of C57 and 129 (*Tbx6^tm1Pa^*) or 129 and ICR (Taconic) (*Tbx6^tm2Pa^*). ICR mice were used for *Tbx6* in situ hybridization (ISH). B6.L-*Tbx6^rv^*/J mice were recovered from cryopreserved embryos (JAX) and mated with C57BL/6Tac mice (Taconic). Mice carrying the *Nodal-lacZ*, *TOPGal* [23], and *TCF/Lef-lacZ* [45] reporter genes were crossed with *Tbx6^tm2Pa^* heterozygotes, and double heterozygotes were backcrossed to *Tbx6^tm2Pa^* heterozygotes. Mice with a null mutation in *Wnt3a* [46](JAX) were crossed with *Tbx6^ tm2Pa^* heterozygotes.

Embryos were collected from timed matings; noon on the day of the plug was considered embryonic day (E) 0.5. Early embryos were staged morphologically [47]; in later embryos, somite numbers were used for staging wild type embryos and mutant embryos were stage matched by head or heart morphology. Genotypes of embryos carrying *Tbx6^tm2Pa^* were ascertained on the basis of fluorescence intensity, which, as confirmed by PCR, correlates with the number of mutant alleles. E7.5–8.5 embryos from other crosses were genotyped by PCR using the following primers pairs (1) 5′-GGGAGAATGAGGATCCAGG-3′, (2) 5′-TACCATCCACGAGAGTTGTAC-3′ to obtain a 200 bp wild type allele fragment; and (3) 5′-ATTGCACGCAGGTTCTCCGG-3′; (4) 5′-GTCACGACGAGATCCTCGCC-3′ to obtain a 550 bp mutant allele fragment. E9.5-10.5 null and compound heterozygous mutant embryos were recognized by the characteristic mutant phenotype [36], [38].

### ISH and β-galactosidase staining

Embryos for ISH were dissected in phosphate buffered saline (PBS) with 0.2% albumin bovine serum (Fraction V, Sigma), fixed overnight in 4% paraformaldehyde in PBS at 4°C, dehydrated in methanol and stored at −20°C. Whole mount ISH was performed using antisense riboprobes [48]. β-galactosidase staining was performed according to standard protocols [49].

#### Immunofluorescence

Embryos were dissected in PBS and fixed for 2–24 hours in 4% paraformaldehyde in PBS at 4°C, washed in PBS and stored in PBS + 0.1% azide at 4°C. The protocol described by Nakaya *et al.* [33] was used with minor modifications. Briefly, after incubation with the primary antibody, embryos were incubated in blocking solution (Vector Labs, Vectastain ABC kit) for 1 h at 4°C, followed by incubation with secondary antibody overnight. Embryos were extensively washed in TBST, incubated with streptavidin-conjugated substrates (e.g. Streptavidin AlexaFluor-543 or Streptavidin AlexaFluor-633, Molecular Probes) for 30 m-1 h at room temperature, and then washed in TBST. Embryos were counterstained with Hoechst (Molecular Probes) to label nuclei, mounted in SlowFade Antifade (Molecular Probes) and imaged using laser scanning confocal microscopy. Primary antibodies used were against acetylated-tubulin (1∶400, Sigma) and Tbx6 (1∶300, Santa Cruz).

#### Scanning Electron Microscopy (SEM)

Embryos for SEM were dissected in PBS and fixed in 0.25% glutaraldehyde in PBS for at least 48 hours, dehydrated into 100% ethanol and critical-point dried prior to coating with gold/palladium in a Denton Vacuum Desk 1V sputter coater. Coated embryos were mounted onto double-sided adhesive tape on metal stubs and photographed with a Jeol JSM 35 Scanning Electron Microscope or a Zeiss Field Emission Scanning Electron Microscope Supra 25.

#### Analysis of Calcium Signaling

For live imaging of calcium signaling, embryos carrying the *Tbx6^tm1Pa^* allele were dissected at E7.5-E8.0 in PB-1 medium [50], then placed in 50% DMEM/F12 and 50% rat serum supplemented with penicillin-streptomycin and glutamine for 30 m prior to incubation with the fluorescent calcium indicators Fluo-3 or Fluo-4 (Molecular Probes; 10 μm in DMEM/F12) with 25% rat serum for 20 m. Fluo-4 is an analog of Fluo-3 with increased fluorescence excitation at 488 nm, thus Fluo-4 was used for all mutant embryos analyzed. Embryos were then washed 3 times in DMEM/F12 with 25% rat serum and placed node-side down into individual wells made in an agarose-coated prewarmed coverslip-bottomed dish (MatTek). The dish was re-equilibriated in an incubator for 10–20 m and then placed in a heated, humidified and gassed chamber on an inverted microscope (Zeiss Axiovert 200M) attached to a Zeiss LSM510 META laser scanning confocal for live imaging. Fluo-3 and Fluo-4 were excited using a 488 nm Argon laser. *z*-stacks of *xy* images were acquired at 2 μm intervals using a 20x/NA0.75 Plan-Apochromat objective. All dissections, incubations and imaging were performed at 37°C. Raw data was processed using Zeiss AIM software (Carl Zeiss Microsystems at http:://www.zeiss.com/).

#### Imaging cilia movement

To generate plasmid *pCX::tau-GFP*, a green fluorescent protein (GFP) fusion to the bovine microtubule binding protein tau with a rabbit β-globin polyadenlyation sequence was placed under the regulation of the *CAGGS* promoter [51], which drives widespread transgene expression. The *CAG::tau-GFP* strain of mice was generated through ES cell mediated transgenesis [52]. Mice having widespread expression are viable and fertile and indistinguishable from non-transgenic littermates (S. Nowotschin and A.-K.H. unpublished observations).

Mice carrying the *CAG::tau-GFP* transgene were crossed with mice carrying the *Tbx6^tm2pa^* allele. Embryos for live imaging of nodal cilia movement were treated as described for Ca^2+^ signaling. GFP was excited using a 488 nm Argon laser. Where necessary linear unmixing [53] was used to separate GFP from yellow fluorescent protein (YFP) as described previously [54]. *z*-stacks of *xy* images were acquired at 1 μm intervals using either a 20x/NA0.75 Plan-Apochromat or a 40x/NA1.3 Plan-Neofluar objective. Scan speeds were set at approximately 3 seconds per *xy* frame, with each frame comprising 512×512 pixels. z-stacks were rendered and time-lapse movies were generated using the Zeiss LSM Image Browser. Cilia were analyzed with the investigator blind to the genotype of the embryos.

## Results

### Heterotaxia of *Tbx6* null mutant embryos

**Figure 1:**
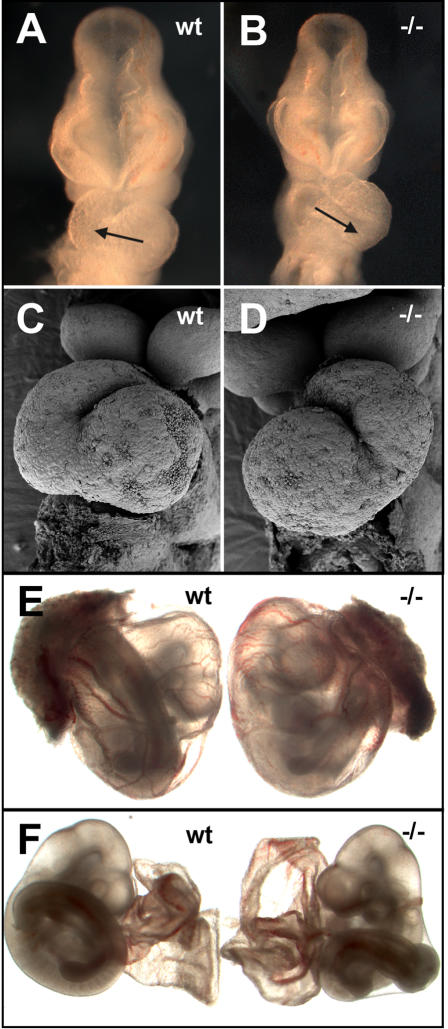
Laterality defects in E9.5 *Tbx6* mutant embryos. (A) Ventral view of a wild type (wt) embryo with normal heart looping and (B) a *Tbx6* homozygous mutant embryo (−/−) with reversed heart looping. The arrow indicates the direction of looping from the inflow to the outflow tract. ( C, D) SEM of the heart of a wild type embryo and mutant embryo with reversed looping, respectively. (E) Embryos still enclosed in the yolk sac with the placenta attached or (F) with the placenta removed and the yolk sac everted. The wild type embryo shows the normal arrangement of placenta, tail, and vitelline vessels, whereas the mutant has reversed orientation.

The dextral looping of the embryonic heart is one of the earliest morphological signs of bilateral asymmetry. Normally, the heart tube loops horizontally from left (inflow) to right (outflow) in a C-shaped loop when viewed from the ventral aspect. In *Tbx6* null mutants, 40% of E9.5-E10.5 embryos (22/55) showed abnormal heart looping that was either completely reversed (J-shaped) or looped ventrally rather than horizontally (Table 1 and Figure 1A–D) whereas all heterozygous and wild type embryos (n = 231) had normal heart looping. Rotation of the mouse embryo, during turning to a fetal position, is normally in an anticlockwise direction (towards the embryo's right side) resulting in the embryo becoming enveloped by its membranes with the umbilical vessels, placenta, and tail on the right side of the embryo (Figure 1E). The vitelline vessels enter and leave the yolk sac on the left side (Figure 1F). While the pattern is rarely disrupted in *Tbx6* heterozygous and wild type mice (4/151; 3%), it is completely reversed in 18% of *Tbx6* homozygous null mutant embryos (7/38), indicating that turning took place with a clockwise rotation (Figure 1E&F). An additional 4 embryos (4/38) showed abnormal laterality of one or more of these features (Table 1).

Reversal of heart looping did not always correlate with the direction of turning, as two mutant embryos exhibited reversed turning but normal heart looping and 8 embryos had reversed heart looping but normal turning. This result indicates heterotaxia, the independent randomization of situs in different features. Overall, 50% (19/38) of mutant embryos scored for heart looping and turning displayed some form of laterality defect. Null/hypomorphic (*Tbx6^tm2Pa^/Tbx6^rv^*) compound heterozygous mutants, on the other hand, did not display any abnormalities in laterality (n = 22, Table 1). These results indicate that the loss, but not the reduction, of *Tbx6* randomizes laterality determination in early embryos.

**Table 1:** Laterality defects in E9.5–E10.5 embryos from crosses between *Tbx6^tm2Pa^/+* and *Tbx6^tm1Pa^/+* heterozygous mice and between *Tbx6^tm2Pa^/+* and *Tbx6^rv^/+* heterozygous mice.

Genotype	Scored for heart only			Scored for heart, placenta, umbilicus, vitelline vessels and tail position								
	n	normal situs	heart reversed	n	normal situs	situs inversus	heart only reversed	placenta, umbilicus, vitelline vessels and tail reversed	tail and vitelline vessels reversed	vitelline vessels reversed	placenta and tail reversed	tail reversed
*Tbx6^tm2Pa^/Tbx6^tm1Pa^*	17	10	7^1^	38	19^2^	7^2^	8^3^	2	1	1	0	0
*Tbx6^tm2Pa^ /Tbx6^rv^*	9	9^4^	0	13	13^2^	0	0	0	0	0	0	0
wild type^5^	80	80	0	151	147	0	0	1	0	0	1	2

1 Two had ventrally looped hearts

2 Two had tails twisted 180^0^ so that the umbilicus looped around the dorsal side of the tail; this appeared to be due to greater flexibility of the tail of mutants because of the lack of somites, rather than to an altered direction of turning

3 Five had ventrally looped hearts

4 One had a tail twisted 180^0^

5 Wild type includes all morphologically normal littermates, which include +/+ and heterozygous embryo

### Molecular correlates of laterality defects in *Tbx6* mutant embryos

**Figure 2:**
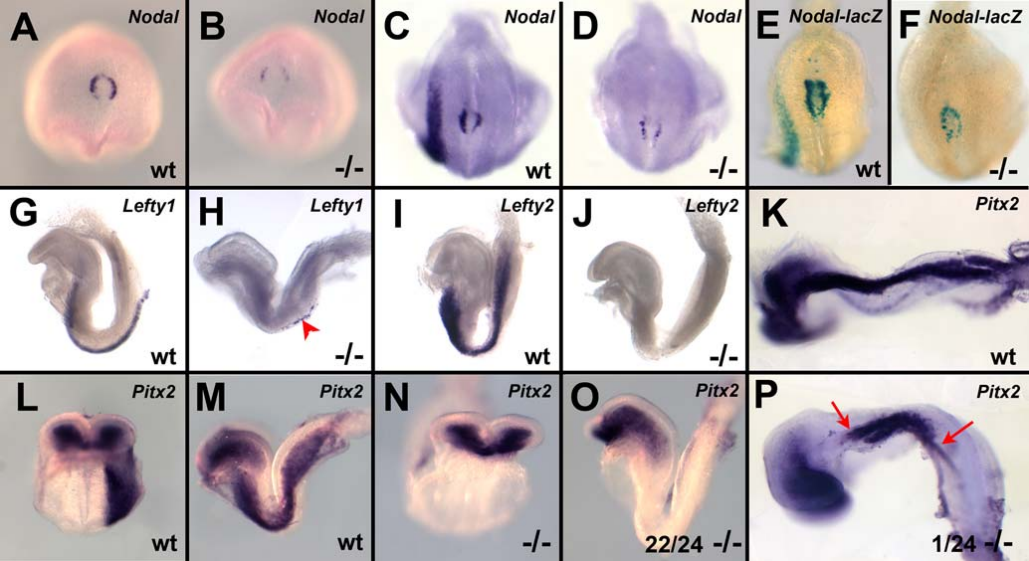
Down regulation of *Nodal* and lack of asymmetric expression of molecular markers in *Tbx6* mutant embryos. (A,B) Ventral view of E7.5 embryos showing down regulation of *Nodal* in *Tbx6* mutant (−/−) compared with wild type (wt). (C,D) Posterior views of the node of E8.0 embryos, showing down regulation of *Nodal* perinodally and no expression in the LPM of mutant embryos compared with left-sided expression in the controls. (E,F) β galactosidase staining in embryos carrying *Nodal-lacZ* shows similar downregulation of *Nodal*. (G,H) Lateral views of *Lefty1* ISH. *Lefty1* was detected throughout the midline in a single wild type embryo (G) and in scattered cells (arrowhead) in the midline in two mutants (H). (I,J). Lateral views showing that *Lefty2* is not expressed in left LPM in E8.0 mutant embryos as it is in wild type embryos. (K–P) Frontal and lateral views of *Pitx2* ISH in E8–8.5 embryos showing no LPM expression in 22/24 mutants at 3–6 somite stages (N,O) but expression in the left LPM of a 7–8 somite stage embryo (P) that is more restricted (arrows) than a comparably staged wild type embryo (K).

*Nodal* expression is normally seen around the node at early gastrulation, becoming stronger on the left side. At 2–6 somite stages, it appears in the left LPM. In *Tbx6* null embryos (n = 6), perinodal expression was reduced at E7.5 in early head fold (EHF) to late head fold (LHF) stages compared with wild type embryos (*Tbx6 +/+* and *+/−*; n = 15)(Figure 2A,B). Decreased expression was also observed in E8.0–8.5 mutant embryos at 3–7 somite stages. Furthermore, none of these mutants showed expression in the LPM (n = 5) whereas robust expression around the node and in the left LPM was observed in wild type embryos (n = 7) (Figure 2C,D). To extend the narrow time window of *Nodal* detection by ISH, we made use of a *Nodal-lacZ* reporter allele [23]. In *Nodal-lacZ* heterozygous embryos of 5–12 somites, β galactosidase staining reported robust *Nodal* expression around the node and in the left LPM of wild type embryos (n = 32) whereas compound *Nodal-lacZ* heterozygous, *Tbx6* mutant embryos equivalent to 3–10 somite stages as judged by head morphology showed only modest expression around the node and no expression in the LPM (n = 8)(Figure 2E,F). This lack of expression, however, could be compounded by the fact that the *Nodal-lacZ* allele is a null allele.

*Lefty1*, which is transiently expressed in the left floor plate of the neural tube at early somite stages, was detected in 1/16 wild type and 3/18 mutant embryos (Figure 2G,H). Two of these mutants had only a few scattered cells in the midline (Figure 2H), whereas the other had expression throughout the length of the floor plate similar to the wild type embryo. *Lefty2* and *Nodal* are normally coexpressed in the left LPM. We found no expression of *Lefty2* in the LPM in homozygous mutant embryos (n = 9) whereas expression was detected in the left LPM or heart of 8/10 stage-matched wild type embryos (Figure 2I,J).

*Pitx2* is a global executor of L/R patterning. It is a target of the nodal and Lefty pathways in the LPM and is expressed shortly after *Nodal* in a broader domain on the left side. At 3–6 somite stages, wild type embryos showed *Pitx2* expression in the left LPM, which became limited to the left inflow tract of the heart during 7–10 somite stages (n = 33). At stages where LPM expression of *Pitx2* would be expected, the majority of homozygous mutants had no LPM expression (22/24)(Figure 2L–O), but a single mutant embryo of ∼3 somites had expression in the right LPM and another of ∼8 somites had expression in the left LPM in a more restricted domain than wild type (Figure 2K,P). At later stages, no mutant embryos had LPM expression (n = 14). Only 4/14 mutant embryos had expression in the left atrium of the heart, 2 of which had dextrally looped hearts. The mutant embryos lacking expression either had hearts that had not yet looped (n = 6) or had ventrally looped hearts (n = 4). Taken together, these results indicate that in the absence of *Tbx6*, *Nodal* expression is greatly diminished around the node and is missing from the left LPM. The absence of either *Nodal* or *Lefty2* in the left or right LPM, the lack of *Pitx2* expression in most mutant embryos, and inappropriate right-sided expression in one mutant indicate that the transfer of laterality information from the node to the left lateral plate is disrupted.

### Alterations in Notch signaling in *Tbx6* mutants

**Figure 3:**
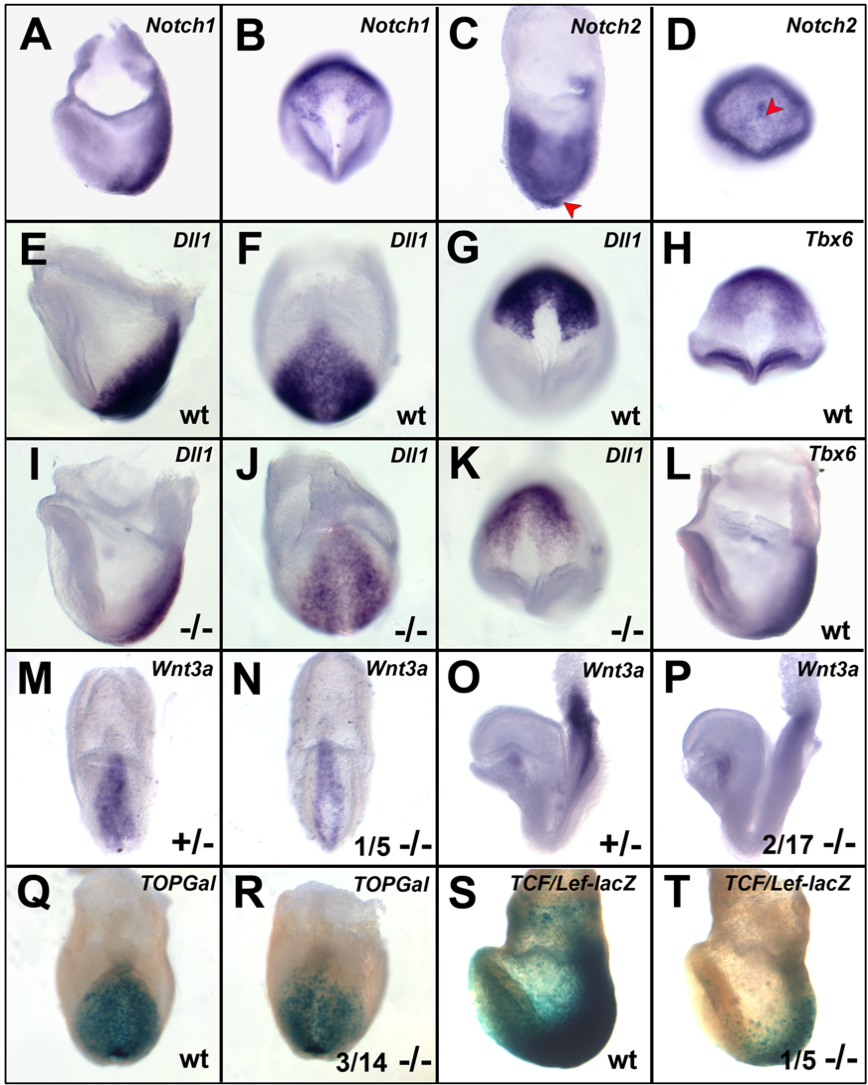
Disruption of some components of the Notch and WNT signaling pathways in *Tbx6* mutant embryos. (A–D) Lateral and ventral views of ISH for *Notch1* and *Notch2* revealed no differences among embryos from *Tbx6* heterozygous crosses. *Notch1* is excluded from the node and *Notch2* is expressed in the node (arrowheads in C and D). (E–G, I–K) Lateral, posterior and ventral views, respectively, of ISH for *Dll1* in wild type (wt) and homozygous *Tbx6* mutant (−/−) embryos reveals decreased expression in mutant embryos. (H,L) Ventral and lateral views, respectively, show that *Tbx6* expression overlaps with that of *Dll1* in the PSM and around the node. (M–P) ISH for *Wnt3a* in heterozygous and homozygous mutant embryos of late bud (M,N, posterior views) and ∼3 somite (O,P, lateral views) stages. Most mutant embryos were similar to wild type, whereas 1/5 and 2/17 mutants showed decreased expression (N,P). (Q,R). Posterior views of β-galactosidase staining in embryos carrying the *TOPGal* transgene reveals a lower level of WNT signaling in 3/14 *Tbx6* mutant embryos whereas the majority were indistinguishable from wild type. (S,T) Lateral views of β-galactosidase staining in embryos carrying the *TCF/Lef-lacZ* transgene shows 1/5 *Tbx6* mutants with reduced WNT signaling, with the remainder similar to wild type.

Notch signaling is known to be upstream of *Nodal* expression in the node. The decreased *Nodal* expression observed around the node implicates the Notch signaling pathway in the disruption of laterality determination in *Tbx6* mutant embryos. We examined the expression of Notch signaling pathway components in *Tbx6* mutant embryos. The Notch receptors *Notch1* and *Notch2* which are both required for the establishment of asymmetry were expressed normally in all E7.5 embryos (n = 17 and 13, respectively) from *Tbx6* heterozygous matings (Figure 3A–D) indicating that there is likely no major disruption of expression of these receptors in mutant embryos. Expression of the Notch ligand, *Dll1*, however, is greatly diminished in the primitive streak and PSM of *Tbx6* mutant embryos at E7.5 (n = 6)(Figure 3E–G, I–K).

**Figure 4:**
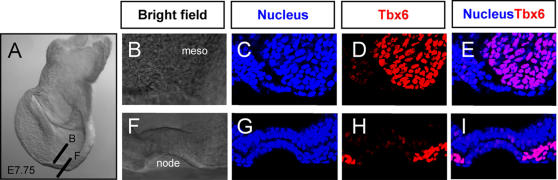
Tbx6 is present in the nascent mesoderm but excluded from the node. (A) Wholemount brightfield image of an E7.75 mouse embryo depicting the region where laser scanning confocal sections depicted in the following panels were acquired. (B–E) Region encompassing nascent mesoderm and neural plate. (F–I) Cross-sectional optical slice through the node. Blue fluorescence, nuclear staining; red fluorescence, Tbx6 immunostaining; pink fluorescence, nucleus and Tbx6 staining.

*Dll1* is a known downstream target of *Tbx6* in the PSM at E8–8.5 [42]. We previously showed that *Tbx6* is expressed in the primitive streak and PSM from E7.0, but is excluded from the node [35]. To compare with *Dll1* expression at E7.5, we examined wild type embryos (n = 8) and found *Tbx6* expression extending halfway around the node in EHF to LHF stage embryos in an area overlapping with *Dll1* expression (Figure 3H,L). Furthermore, we have shown that Tbx6 protein is present in the PSM but not in the node (Figure 4). This suggests that *Tbx6* expression overlaps with *Dll1* in the PSM and perinodal region at the time L/R asymmetry is established and that the reduction in *Dll1* expression in *Tbx6* mutant embryos could be a direct effect.

### WNT signaling is marginally affected in Tbx6 mutant embryos

Because WNT signaling in the node regulates nodal signaling through the Notch pathway [33], we assessed WNT signaling in *Tbx6* mutant embryos. In early bud to LHF stage embryos, *Wnt3a* expression in the primitive streak was similar between wild type (n = 7) and 4/5 *Tbx6* mutants; the exceptional mutant embryo showed less intense expression (Figure 3M,N). In embryos ranging from 1–12 somites, *Wnt3a* expression in PSM, neural plate and the base of the allantois was similar in wild type (n = 6) and mutant (n = 17) embryos, although 2 mutants of approximately 3–6 somites had reduced expression compared with stage matched controls (Figure 3 O,P).

To determine sites of active WNT signaling, we used two transgenic lines, *TCF/Lef-lacZ* and *TOPgal* that report Wnt/β-catenin activity with β–galactosidase readout. With *TOPgal*, β–galactosidase activity was detected in both *Tbx6* wild type (n = 24) and homozygous mutant (n = 14) embryos, although 3 mutants had reduced levels in the PSM (Figure 3Q,R). With *TCF/Lef-lacZ*, 1/5 mutant embryos had reduced PSM expression compared with wild type (n = 8; Figure 3S,T). Thus most mutant embryos are indistinguishable from wildtype, although a small proportion (7/41; 17%) had reduced *Wnt3a* expression or WNT signaling.

To determine whether *Tbx6* and *Wnt3a* interact genetically, we intercrossed *Tbx6^tm2Pa^* and *Wnt3a* heterozygotes and examined the direction of heart looping and somite morphology of double heterozygous offspring. At E10.5, all *Tbx6; Wnt3a* double heterozygotes (n = 18) and all *Tbx6* single heterozygotes (n = 19) displayed normal heart looping and somite formation, indicating that there is no genetic interaction between *Wnt3a* and *Tbx6* with respect to direction of heart looping or somite segmentation. We conclude that although some *Tbx6* homozygous mutants display reduced Wnt signaling, this may not play a significant role in the disruption of laterality determination in *Tbx6* mutant embryos.

### Abnormalities of the node and nodal cilia

**Figure 5:**
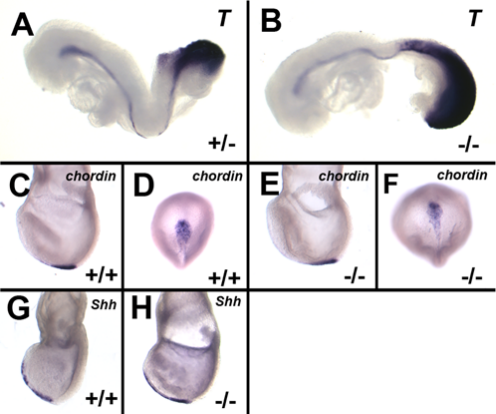
Expression of midline and node markers is not disturbed in *Tbx6* mutant embryos. (A,B) *Brachury* expression in the notochord and presomitic mesoderm at E8.5, (C–F) *Chordin* expression in the node at E7.5 (lateral and ventral views), and (G–H) *Shh* expression in the node and anterior midline at E7.5 is similar between wild type (+/+ or +/−) and *Tbx6* mutant (−/−) embryos.

The integrity of the midline, node and nodal cilia was examined using additional molecular markers and by direct observation of the nodal cilia. We saw no indication of disruption of the notochord in embryos of 2–8 somites using a *Brachyury* probe (n = 5 wildtype; 8 mutants) or of the node and anterior midline of E7.5 embryos using *chordin* (n = 17 wild type; 6 mutants) or *Shh* probes (n = 7 wild type, 5 mutants) (Figure 5).

**Figure 6:**
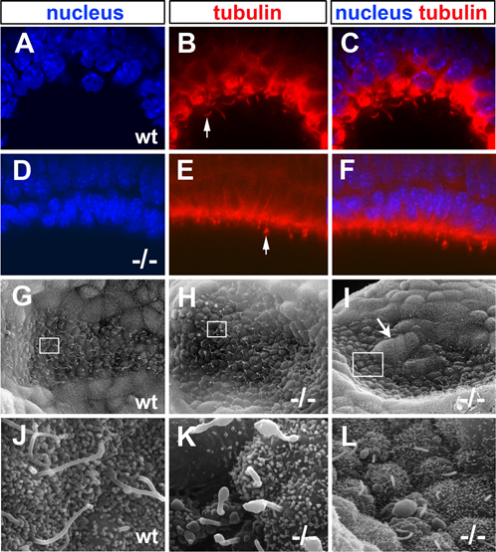
Nodal cilia are present but abnormal in *Tbx6* mutant embryos. (A–F) Confocal images through the node of wild type (wt) and *Tbx6* mutant −/−) embryos stained with anti-acetylated tubulin with a Hoechst nuclear counterstain. Cilia visible on the ventral surface are filamentous in wild type but are short with bulbous tips in the mutant. (G–L) SEM images of the node and nodal cilia in wt and −/− nodes (G,J) Normal, long filamentous cilia in wild type nodes. (H,K) Mutant node showing cilia with blebs and bulbous tips typical of most mutants. (I,L) Atypical mutant node with short cilia and an abnormally shaped node with large visceral endoderm-like cells within the node (arrow). The boxes in G–I correspond to the higher magnification views in J–L.

Antibodies against acetylated α-tubulin, which marks stable microtubules, were used to detect the presence of cilia in the node of embryos at E7.5–8.0. Wild type embryos (n = 4) had long, filamentous nodal cilia (Figure 6A–C) whereas homozygous mutant embryos (n = 6) showed evidence of shorter, thicker cilia, some with terminal knobs (Figure 6D–F). SEM revealed gross abnormalities in the monocilia of the node in *Tbx6* mutant embryos (n = 15) compared with wild type embryos (n = 6). Wild type cilia were long slender projections (Figure 6G,J) whereas most mutant cilia had terminal bulges or balloon-like structures (Figure 6H,K). A single mutant embryo had straight cilia but these were shorter than wild type (Figure 6I,L). The node of this embryo was also misshapen and contained cells with the appearance of the surrounding visceral endoderm. (Figure 6I). Clearly, the lack of *Tbx6* has a profound effect on the morphology of monocilia in the cells of the node, even though *Tbx6* is not expressed in these cells at this time.

### Live imaging of nodal cilia motility

**Figure 7:**
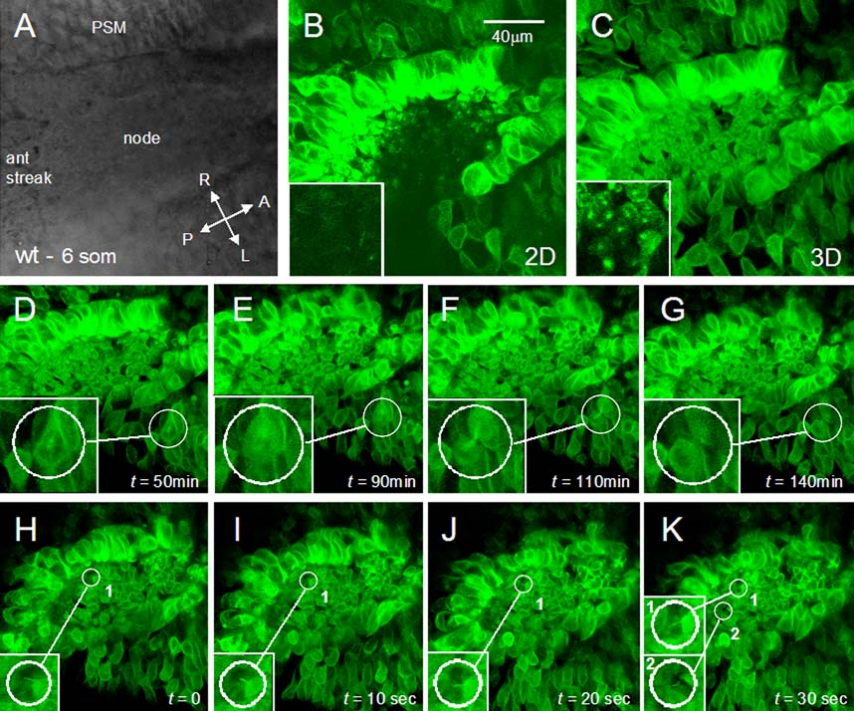
A widely expressed *tau-GFP* fusion for imaging microtubule dynamics in live mouse embryos. *CAG::tau-GFP* hemizygous embryos were cultured and imaged ventrally with the node close to the objective. (A) Brightfield image of the perinodal region of a 6 somite embryo. (B) Single laser scanning (*xy* = 2D) optical slice taken from a *z*-stack. Inset of region within pit of node reveals elongated GFP+ structures that resemble cilia. (C) 3D reconstruction of *z*-stack acquired at the perinodal region. Inset of a region within the pit of the node reveals the position of basal bodies of cells. (D–G) Single frames from a 3D time-lapse sequence acquired in the perinodal area reveal cell division dynamics. Each frame represents a 3D reconstruction of a *z*-stack taken at indicated time-points. Insets depict the dynamic reorganization of microtubles in a paraxial mesoderm cell undergoing division. Interphase (D), anaphase (E) and cytokinesis (F and G). (H–K) Single frames from high-speed 3D time-lapse sequence acquired in the perinodal area reveal the change in position of elongated GFP+ cilia (insets) over time.

To determine if the cilia of *Tbx6* mutant embryos are motile, we devised a method to directly image cilia in living embryos that relies on 1) a strain of mice expressing a fluorescent protein fusion that labels cilia, and 2) a mode of image acquisition that provides information on the dynamics of GFP distribution thereby providing a readout of cilia movement. We reasoned that we could non-invasively label and subsequently live image cilia by imaging tubulin, a central structural component of cilia, using genetically-encoded fluorescent protein fusions that would incorporate into microtubules. We generated a fusion of GFP to the bovine microtubule binding protein tau and demonstrated that it labeled and permitted the dynamic visualization of microtubules in embryonic stem (ES) cells and mouse embryos, most easily observed through stereotypical rearrangements in the cytoskeleton that accompany mitosis (Figure 7D–G and supplementary Video S1).

Laser point scanning confocal optical sections in the vicinity of the node reveal fluorescence in basal bodies of nodal cells connected to long, narrow projections. These are easily seen in single (*xy*) optical sections taken from a *z*-stack of the entire node (Figure 7A–C and supplementary Video S1). To confirm that we were visualizing cilia and to document their movement, high-speed acquisition of *z*-stacks of *xy* sections were taken at timed intervals (3D time-lapse). Subsequent 3D rendering of *xyz* data revealed GFP-labeled projections that changed their orientation between successive time points (Figure 7H–K), suggesting movement and supporting the idea that the observed projections are cilia. However, since it has been determined that nodal cilia rotate at approximately 600 rpm [17], the rate of image acquisition possible with either a spinning disc or laser scanning confocal is too slow to permit the continuous imaging required to visualize rapid rotation.

**Figure 8:**
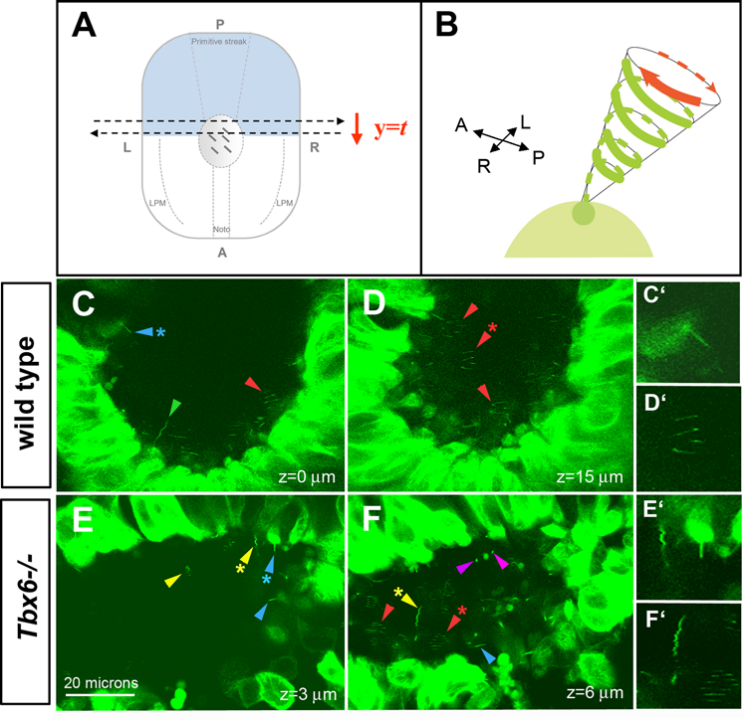
Imaging cilia motility in *Tbx6* wild type and mutant embryos using a *tau-GFP* transgene. (A) Schematic representation of the direction of scanning with time to obtain images of nodal cilia in living embryos. (B) Schematic representation of the GFP positive trace that would be left by a regularly rotating cilium. (C–F) Live imaging cilia motility in the nodes of *Tbx6* wild type and *Tbx6* mutant (−/−) embryos at E8, visualized by laser scanning confocal microscopy. Straight (blue arrowheads) or wavy (green arrowhead) traces, mostly at the periphery of the nodes (C,C′,E,E′), indicate stationary or slowly moving cilia in both wild type and mutant nodes. Regular beat, whip-like, sinusoidal traces (red arrowheads), most evident deeper in the pit of the nodes (D,D′,F, F′), indicate cilia with rapid regular rotation in both wild type and mutant nodes. In addition, mutant embryos (E, F) display erratic, irregular zigzag traces (yellow arrowheads) and stationary or moving spots (purple arrowheads) indicating cilia with abnormal movement and/or morphology. Asterisks indicate the cilia shown in the blown up details C′, D′, E′, F′.

To circumvent this problem, we acquired *z*-stacks of optical slices (i.e. *xy* images) of the node with a laser point scanning confocal microscope at a defined speed (determined empirically to be between 3 and15 s) to generate kymographs that depict cilia movement. In such optical sections, the *y*-axis represents a time axis (Figure 8A) and the *xy* image serves as a kymograph documenting the spatial displacement of GFP revealed by the traces of GFP fluorescence reflecting cilia movement (Figure 8B). Because we acquired *z*-stacks of *xy* data the majority of cilia were imaged on consecutive optical sections representing independent measurements and verification of their movement. We observed a variety of types of GFP traces which we have grouped into 5 categories (Table 2): 1) regular beat: sinusoidal traces or a series of parallel lines were interpreted as motile cilia rapidly rotating with constant periodicity, the shape of the trace depending on the angle of observation, (Figure 8B,C,D,F, red arrowheads); 2) straight: nonmotile cilia were observed as straight lines that did not move or moved only slightly between consecutive sections (Figure 8C,E,F, blue arrowheads); 3) wavy: wavy traces that moved slightly between consecutive sections suggested slowly swaying cilia that either could be weakly motile cilia or nonmotile cilia being passively moved by fluid currents (Figure 8C, green arrowhead; 4) erratic: erratically moving, zigzag traces indicated motile cilia with uncoordinated, irregular movements (Figure 8E,F, yellow arrowheads); 5) spots: stationery or moving spots of fluorescence could either indicate morphologically abnormal short cilia or immotile cilia viewed along their length.

### Abnormal cilia motility in *Tbx6* mutant embryos

Using this new method of visualizing cilia motility in living embryos, we observed that all wild type embryos (n = 13) displayed mainly two types of traces, 1) regular beat, corresponding to motile cilia (Figure 8C,D,D′, red arrowheads) and 2) straight, corresponding to nonmotile, presumably mechanosensory cilia (Figure 8C,C′, blue arrowhead), with minor populations of cilia in the other categories described in the previous section. The proportion of each type of cilia at the periphery and in the pit of the node was quantified for 8 wild type embryos for which the *z* stacks encompassed the node from crown to pit. At the periphery of the node, nonmotile cilia predominated (57%) whereas regularly beating cilia predominated in the pit of the node (65%) but were also fairly common at the periphery (18%, Table 2). Cilia categorized as wavy were more common at the periphery than in the pit of the node, compatible with these being nonmotile, mechanosensory cilia.

**Table 2:** Classification of cilia at the periphery and in the pit of the node from 8 wild type and 4 mutant embryos for which *z*-stack images encompassed the entire node.

				Number of cilia (%)				
Genotype	Region of node	No. of embryos	No. of cilia	Straight	Wavy	RegularBeat	Erratic	Spots
Wild type	Periphery	8	288	165 (57)	34 (12)	51 (18)	18 (6)	20 (7)
	Pit	8	197	30 (15)	10 (5)	128 (65)	13 (7)	16 (8)
Mutant	Periphery	4	129	73 (57)	7 (5)	11 (9)	20 (16)	18 (14)
	Pit	4	153	51 (33)	7 (5)	38 (25)	24 (16)	33 (22)

*Tbx6* mutant embryos (n = 6) displayed the same types of cilia but the proportion of each type and the distribution between the periphery and the pit of the node was significantly different from wild type in the 4 embryos with images spanning from the crown to the pit of the node (Table 2) (Χ^2^ = 21.8, 4df, p<001 for distribution at the periphery; Χ^2^ = 59.4, 4df, p<0.001 for distribution in the pit). At the periphery of the node in mutants, the proportion of straight cilia (Figure 8E,E′, blue arrowheads) was the same as wild type (57%) but there was a lower proportion of waving and regularly beating cilia and higher proportions of erratically moving cilia (Figure 8E,E′ yellow arrowheads) and spots, which most likely represent the short, globular cilia observed with SEM and anti-tubulin (Figure 6). In the pit of the node in mutants there were higher proportions of nonmotile cilia (Figure 8F, blue arrowhead), erratically moving cilia (Figure 8E,E′,F,F′; yellow arrowheads) and spots (Figure 8F, purple arrowheads) compared with controls, and a decrease in the proportion of regularly rotating cilia (Figure 8F,F′, red arrowheads). These results indicate that *Tbx6* mutant embryos display severe ciliary defects throughout the node but particularly in the pit of the node with the proportion of regularly rotating cilia decreased at the expense of erratically moving or stationary cilia, many of which have abnormal morphology.

Furthermore, the *tau-GFP* transgene, which highlights microtubules, revealed cellular irregularities within and around the node of mutant embryos (Figure 8E,F). The movement of cilia and the differences between the cellular structure of *Tbx6* mutant and wild type nodes can be visualized in the movies (*z* stacks) of successive images (Supplementary Videos S2 and S3).

### Loss of Tbx6 results in loss of asymmetric intracellular Ca^2+^ at the periphery of the node

**Figure 9:**
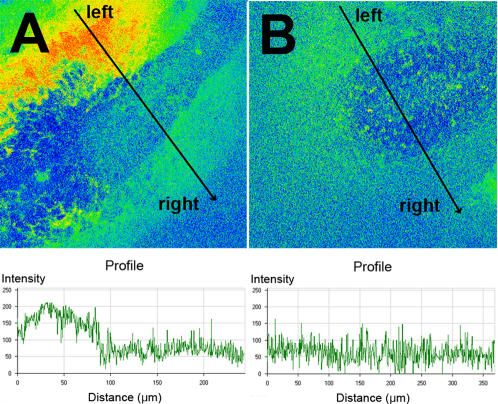
Calcium signaling at the periphery of the node as visualized by confocal microscopy of the calcium ionophore, Fluor-4 in wild type (A) and *Tbx6* mutant (B) embryos. The arrows indicate the position and direction of the trace below each image measuring the intensity of fluorescence from the left side to the right side of the node. The asymmetric Ca^2+^ signal seen in wild type embryos is completely absent in *Tbx6* mutant embryos.

Asymmetric Ca^2+^ has been detected on the left side of the node both extracellularly in chick [31] and intracellularly in mouse [19] and is dependent on a leftward nodal flow. In order to determine whether the abnormalities in the node and nodal cilia in *Tbx6* mutant embryos affects Ca^2+^ signaling, we incubated whole embryos (yolk sac and placenta removed) in the presence of either the Fluo-3 or Fluo-4 ionophores, and imaged whole, living embryos through the node and perinodal region using confocal microscopy. In wild type embryos, we did not detect asymmetric Ca^2+^ levels within cells of the node itself. However in 0–4 somite stage wild type embryos, we observed a robust asymmetric increase of intracellular Ca^2+^ on the left side of the node, which is not present in embryos of 8 somites or greater. In contrast, *Tbx6* homozygous mutant embryos showed no asymmetries in intracellular Ca^2+^ in the region around the node (Figure 9 and Table 3). As functional cilia and a leftward nodal flow are necessary to generate asymmetric Ca^2+^ around the node [19], these results indicate that the abnormally shaped cilia in *Tbx6* mutant embryos are unable to generate a unidirectional nodal flow.

**Table 3:** Perinodal calcium signaling in intact, living wild type and *Tbx6* homozygous mutant embryos at 0–4 somite stages, visualized with calcium ionophores by confocal microscopy in complete *z*-stacks through the embryo.

*Tbx6* genotype	n	Ca^2+^ on left	Ca^2+^ absent
+/+	8	6	2
+/−	6	5	1
−/−	9	0	9

## Discussion

### *Tbx6* affects Notch signaling around the node

Our study has revealed a novel role for the T-box transcription factor Tbx6 in determination of the L/R body axis. Homozygous mutants, but not compound null/hypomorphic heterozygotes, show randomization of the direction of heart looping and independent alterations in the direction of embryo turning. The molecular phenotype of the *Tbx6* mutant embryos indicates a disruption of nodal signaling and is similar to the phenotype of embryos with decreased levels of *Nodal* expression around the node [26]. We observed no *Nodal* expression in the LPM mesoderm of mutant embryos, however, the downstream target, *Pitx2*, was expressed in the LPM in a minority of mutant embryos. This is most likely due to low residual levels of *Nodal* expression in the mutants as several other mutations with laterality defects show a similar expression of *Pitx2* in the absence of detectable LPM *Nodal* expression [55], [56]. Another possibility is that the LPM itself is missing or defective in *Tbx6* mutant embryos although morphological and molecular evidence argues against this possibility [36](D. Concepcion and V. E. Papaioannou, unpublished observations).

It is known that perinodal nodal signaling is controlled by the Notch signaling pathway and that disruption of *Dll1* or other components of the Notch pathway result in abnormal L/R determination [29]–[32]. Furthermore, as *Dll1* is a direct downstream target of *Tbx6* in the paraxial mesoderm [40], [41], our observation that *Dll1* is down regulated around the node at E7.5 in an area coincident with *Tbx6* expression indicates that *Tbx6* has a direct effect on *Dll1* expression and suggests one possible mechanism for the disruption of L/R determination in *Tbx6* null mutants. The asymmetric expression of *Nodal* is thought to be a response to preexisting asymmetry around the node [31], although no gene with earlier asymmetric expression has yet been found in mice. We do not think that *Tbx6* plays this role but rather that the lack of *Tbx6* results in reduction of *Dll1* and, consequently, *Nodal* expression below a critical threshold for nodal signaling to the left LPM.

The effect of *Tbx6* on *Dll1* expression and the subsequent reduction in *Nodal* could account for the observed disruptions in L/R determination. Three studies of *Dll1* null mutants all report randomized laterality although they disagree as to whether LPM expression of genes such as *Nodal* and *Lefty2* are randomly expressed [32] or absent from the LPM [29], [30], [32], as seen in *Tbx6* mutants. A difference between *Dll1* and *Tbx6* mutants is the expression of *Lefty1* in the latter. Although we saw *Lefty1* in only a single wild type embryo, possibly due to the transient nature of expression, we nonetheless observed expression in three mutants, whereas *Dll1* null mutants have no *Lefty1*. This *Lefty1* expression in *Tbx6* mutants may be due to residual *Dll1* and *Nodal* expression.

*Wnt3a*, which is expressed in the node and PSM, has been implicated in L/R patterning through an effect on the Notch/Delta pathway [33]. Furthermore, there is evidence that *Dll1* is synergistically regulated by *Tbx6* and WNT signaling, and it has been suggested that *Wnt3a* is upstream of *Tbx6* [42]. However, *Tbx6* is normally expressed in *Wnt3a* mutants at early stages (0–2 somites) and is only moderately down regulated later [57]. Thus, *Tbx6* does not appear to be regulated by *Wnt3a* at the time of laterality determination. Because *Tbx6* and *Wnt3a* are expressed in overlapping domains in the PSM, we examined WNT signaling in *Tbx6* mutant embryos and although a few mutants had decreased levels of WNT signaling, the significance of this observation is not clear. We also investigated whether *Tbx6* and *Wnt3a* genetically interact but found no disruption of laterality in double heterozygotes. Thus, the inactivation of one allele of each gene is not sufficient to disturb laterality determination. It is possible that a more sensitized screen using hypomorphic and null alleles might reveal a genetic interaction.

### New method of live imaging cilia and cilia movement

We have developed a new method of visualizing the movement of cilia of the node in living mouse embryos. The *tau-GFP* transgene produces a fusion protein that marks tubulin and allows the visualization of microtubules, including basal bodies and cilia. Using this transgene with scanning confocal microscopy at a reduced scan speed, we have produced kymographs of cilia, essentially tracing their movement in real time. We have visualized the relatively stationary mechanosensory cilia around the periphery of the node, as well as the regular, rapid rotational movement of nodal cilia. Moreover, since the parameters (e.g. the number of pixels per frame and the scan rate) of image acquisition are known, the rotation frequency can be calculated. Interestingly, live imaging of basal bodies in early somite stage (E8.0) wild type embryos did not reveal a caudal bias in their position as has been suggested [5]. Additional experiments will be required to determine if asymmetric positioning of the basal body within nodal cells occurs at earlier or later stages. With this method, we have been able to detect distinct differences in cilia morphology and motility associated with the *Tbx6* mutant phenotype as well as detecting differences in the microtubule structure of the nodal cells themselves. This method will be valuable in making detailed assessments of cilia function in other mutants as well as quantifying the kinetics of movement.

### Effect of *Tbx6* on the node and nodal cilia

Although a decrease in *Dll1* signaling would be sufficient to result in disruptions in L/R axis determination, we discovered that the *Tbx6* mutants also have grossly malformed nodal cilia, a striking departure from the *Dll1* phenotype. In *Dll1* mutants, nodal cilia were normal although disruptions in the structure of the node were noted in a few cases [29], [32]. The cilia morphology of *Tbx6* mutants is similar to that seen in cilia of Chlamydomonas mutants associated with intraflagellar transport (IFT) defects [58], [59]. Moreover, mice with a mutation in *Dnchc2*, encoding a subunit of the retrograde IFT motor [60] show a single bulge along their length. Using a novel method of visualizing cilia movement, we have determined that the morphological abnormalities in the *Tbx6* mutant cilia are accompanied by disruptions in cilia motility. Although we cannot judge whether the short cilia which appear as fluorescent spots are rotating regularly, there are clearly more cilia with erratic movements and more nonmotile cilia in the pit of the node of mutant embryos,. Furthermore, we show that the asymmetric Ca^2+^ flux on the left side of the node is abolished in mutants. It has been demonstrated that asymmetric Ca^2+^ signaling is dependent on a unidirectional nodal flow, as embryos lacking motile cilia do not show elevated Ca^2+^ levels [19]. Thus, using this functional readout of the nodal flow, we favor the interpretation that the cilia of *Tbx6* mutant embryos, some of which are capable of movement, cannot generate a robust unidirectional nodal flow, although we cannot rule out the possibility that the mechanosensory cilia are defective in responding to a flow.

**Figure 10:**
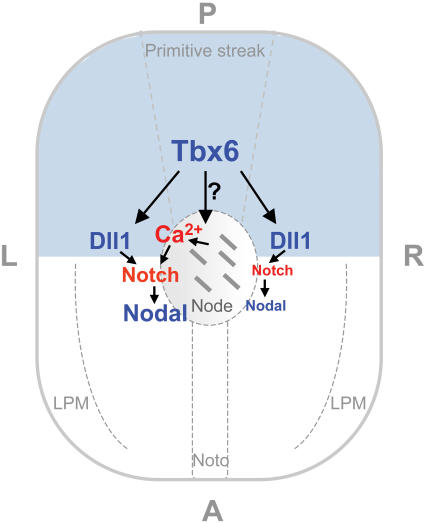
Model of two routes by which Tbx6 could affect left/right axis determination. Symmetric *Tbx6* expression in the presomitic mesoderm and primitive streak (blue area) directly regulates *Dll1* expression, which in turn regulates *Nodal* through notch signaling. In addition, through an unknown mechanism, Tbx6 affects the morphology and motility of the cilia in the node. The model proposes that in the absence of Tbx6, Dll1 and Nodal expression are reduced below a threshold level to initiate the cascade of asymmetric gene expression, and in addition, the motility of the cilia is compromised to the extent that the nodal flow and/or the mechanosensory cilia are disrupted resulting in the lack of Ca^+2^ signaling at the periphery of the node. LPM = lateral plate mesoderm, noto = notochord.

Although it is not known precisely how the nodal flow is translated, one model proposes that mechanosensory cilia at the periphery of the node transduce the nodal flow leading to asymmetric Ca^2+^ signal on the left side of the node [19], [20]. A model has been proposed from evidence in chick that Ca^2+^ modulates Notch signaling activity [31]. Thus, *Tbx6* appears to have effects on at least two separate aspects of laterality determination which could both impinge on Notch signaling: one a direct effect on expression of *Dll1* in PSM and the second, an effect on the morphology and motility of nodal cilia which interferes with the nodal flow (Figure 10).

There appears to be no ortholog of mammalian *Tbx6* in zebrafish [61], [62], but a gene in the same subfamily, *tbx16*, which is mutated in the zebrafish *spadetail* (*spt*) mutation, also has an effect on laterality determination, possibly through multiple pathways similar to what we are postulating for *Tbx6*. *tbx16* regulates the expression of *pkd2*, the gene coding for polycystin-2 (PC-2), the proposed Ca^2+^ ion channel associated with nodal cilia [12] and it regulates nodal activity and notch signaling through regulation of the *nodal* antagonist, *charon* [63].

*Tbx6* and *tbx16* show similarities in expression in PSM and both have effects on somitogenesis. However, *Tbx6* is not expressed in the mouse node whereas *tbx16* is expressed in the precursor cells of Kupffer's vesicle, a ciliated epithelial structure analogous to the mouse node, and is necessary for its morphogenesis [64]. Another T-box gene, *Brachyury*, which is expressed in the node, affects laterality determination in mice [65], as does the ortholog in zebrafish [66]. The *brachyury* mutant *notail* (*ntl*) results in down regulation of the IFT gene, *polaris* [12], and along with *tbx16*, *brachyury* regulates nodal activity and the morphogenesis of Kupffer's vesicle. Thus, while several T-box genes play roles in L/R patterning, *Tbx6* appears to be unique in doing so through an effect on the morphology and function of the nodal cilia in the absence of node expression. It is not known at present how *Tbx6* affects the nodal cilia but it is interesting to note that a peripheral effect on asymmetric gene expression at the node in chick embryos was postulated when it was demonstrated that the induction of asymmetric *Nodal* expression requires secondary signals produced in the PSM [67]. Alternatively, the affected cells of the node might be the progeny of cells that once expressed *Tbx6* and then downregulated *Tbx6* as they migrated into the node. If the node is derived from cells that previously expressed *Tbx6*, the effect on cilia might be through direct transcriptional regulation of genes required for ciliogenesis. Future studies will be needed to trace the lineage of *Tbx6* expressing cells and to identify targets of *Tbx6* in the PSM to make the link between expression of *Tbx6* and the abnormal cilia in the node.

## Supporting Information

Video S3 *z*-stack of *xy* images in the vicinity of the node of an E8.25 (6 somite stage) *CAG::tau-GFP* hemizygous embryo that is homozygous null mutant for *Tbx6*. Starting ventrally at a level around the crown of the node, successive *xy* optical slices are dorsalward and depict regions deeper into the node ending in the pit. The movie contains 34 individual *xy* slices taken at 1 mm intervals.
(0.66 MB MPG)

Video S1 3D time-lapse sequence of a ventral region encompassing the node of an E8.25 (6 somite stage) *CAG::tau-GFP* hemizygous mouse embryo. Dynamics of cell movement and microtubule organization are evident. During the course of the movie several cells located at the periphery (crown) of the node are seen to divide. The movie covers a 4 hr 10 min period. It comprises 50 *z*<-stacks acquired at 5 min intervals. Each frame represents a 3D reconstruction of a 14 mm *z*-stack with each *xy* slice taken at a 2 mm interval.
(0.94 MB MPG)

Video S2 *z*-stack of *xy* images in the vicinity of the node of an E8.25 (6 somite stage) *CAG::tau-GFP* hemizygous mouse embryo, wild type for Tbx6. Starting ventrally at a level around the crown of the node, successive *xy* optical slices are dorsalward and depict regions deeper into the node ending in the pit. The movie contains 25 individual *xy* slices taken at 1 mm intervals.
(0.35 MB MPG)
